# Impact of Bowel Resection on Operative Mortality and Overall Survival in Advanced Epithelial Ovarian Cancer

**DOI:** 10.3390/cancers17132086

**Published:** 2025-06-22

**Authors:** Özgür Ozan Ceylan, İlyas Turan, Evrim Erdemoglu, Marina Santos González, Javier Magrina

**Affiliations:** 1 Division of Gynecologic Oncology, Suleyman Demirel University, Isparta 32200, Turkey; ozgurozanceylan@gmail.com (Ö.O.C.); ilyasturan04@gmail.com (İ.T.); 2 Department of Medical and Gynecologic Surgery, Mayo Clinic, AZ 85054, USA; marinasago@gmail.com (M.S.G.); jmagrina@mayo.edu (J.M.)

**Keywords:** epithelial ovarian cancer, cytoreductive surgery, bowel resection, overall survival, interval debulking, optimal cytoreduction

## Abstract

**Background/Objectives**: Bowel resection may be necessary during cytoreductive surgery (CS) in advanced epithelial ovarian cancer to achieve complete tumor removal. However, concerns about increased perioperative risks and unclear survival benefits have led to ongoing debate. This study aimed to evaluate the impact of bowel resection on perioperative mortality and overall survival (OS) in patients undergoing CS. **Methods**: We retrospectively reviewed 127 patients with FIGO stage IIB–IV epithelial ovarian cancer who underwent primary or interval CS between 2007 and 2021. Patients were stratified based on the performance of bowel resection. Clinical, surgical, and survival data were analyzed using Kaplan–Meier survival analysis and Cox proportional hazards modeling. Primary outcomes were 90-day mortality and OS. **Results**: Bowel resection was performed in 58 patients (46%) with more extensive disease and poorer ECOG performance scores. Although the resection group had increased perioperative risks (e.g., higher transfusion rates and ICU use), OS was similar between groups (log-rank *p* = 0.122). Multivariate analysis identified that increasing age (HR = 1.042, *p* = 0.005) was independently associated with poorer OS, whereas lymph node dissection (HR = 0.450, *p* = 0.003) and undergoing primary CS (HR = 0.540, *p* = 0.047) were associated with improved survival. Bowel resection was not independently associated with OS. **Conclusions**: Bowel resection does not adversely affect OS when optimal cytoreduction is achieved. Although it increases perioperative complexity, it can be safely incorporated into CS in selected patients. These findings support its use as part of an individualized surgical strategy for advanced ovarian cancer.

## Simple Summary

Advanced epithelial ovarian cancer is often diagnosed at a late stage and may require aggressive surgical procedures to improve patient outcomes. One such procedure is bowel resection, which is sometimes needed to achieve complete tumor removal during cytoreductive surgery. However, the necessity and benefit of bowel resection have been debated due to its potential risks. In this study, we compared patients who underwent bowel resection with those who did not, in terms of survival and surgical outcomes. Despite having more extensive disease and requiring more complex operations, patients who underwent bowel resection had similar overall survival rates. Our results suggest that when complete cytoreduction is possible, bowel resection does not negatively affect long-term outcomes. These findings support the use of bowel resection as a part of cytoreductive surgery in carefully selected patients, and highlight the importance of surgical planning and experience in the management of advanced ovarian cancer.

## 1. Introduction

Ovarian cancer is one of the three most common gynecological cancers [1]. Advanced ovarian cancer is the leading cause of death from gynecologic malignancies [1,2,3]. Three-quarters of women with epithelial ovarian carcinoma present with stage IIIC or stage IV disease [2], often with spread to the peritoneum, small bowel, colon, and upper abdomen. Bowel metastasis is the most prevalent among these and is usually accompanied by upper abdominal disease in advanced stages. The most significant, and the only modifiable, factor for survival is the extent of residual tumors following front-line surgery. Neoadjuvant chemotherapy (NACT) followed by interval debulking (IDS) is an alternative approach in women with high-volume disease or stage IV disease, and in women with poor performance scores [4]. The standard of care involves cytoreductive surgery (CS), which aims to remove all visible disease, followed by platinum-based chemotherapy. To achieve maximal cytoreduction of abdominal, pelvic, and retroperitoneal disease, complex operations [5], including bowel resection, may be required during either primary or interval CS [6].

Other studies in the literature have consistently identified postoperative residual tumors as the most significant prognostic factor for survival in patients with advanced ovarian cancer [7,8]. Therefore, there has been a growing endorsement for the implementation of increasingly radical surgical techniques and extensive cytoreduction procedures to maximize the removal of residual tumors. When the disease extensively involves the diaphragm, liver, spleen, pancreas, stomach, or intestine, complex surgical procedures are required to complete the surgery without leaving gross residual disease. It has been previously reported that tumor biology plays a greater role in determining outcomes in advanced disease compared to surgical treatment or adjuvant chemotherapy [7,8], and that bowel resection may be associated with higher perioperative complications, mortality, and worse overall survival [9].

The indications for performing a bowel resection as part of the primary cytoreductive surgery for epithelial ovarian cancer encompass two main objectives: achieving optimal cytoreduction status and managing potential instances of intestinal obstruction [10]. Nevertheless, there is a lack of agreement regarding the optimal degree of surgical resection that should be undertaken by the surgeon to attain complete cytoreduction [3].

The primary aim of this investigation is to evaluate the impact of bowel resection, performed as part of CS, on perioperative mortality and overall survival in patients receiving either primary or neoadjuvant chemotherapy. We focused on mortality and did not include morbidity as part of the primary aim. As a secondary objective, we assessed transfusion requirements, operative time, and postoperative intensive care utilization.

## 2. Materials and Methods

### 2.1. Study Design and Ethical Approval

The study was approved by the Institutional Review Board (protocol ID:270/2022). Patients with pathologically confirmed stage IIB-IV epithelial ovarian cancer undergoing primary or IDS between September 2007 and November 2021 were included.

### 2.2. Patient Selection

Patients with borderline or non-epithelial ovarian tumors and those unfit for surgery were excluded. NACT was preferred in patients with extensive intraabdominal tumor burden, portal hilus invasion, mesenteric vascular involvement, bowel obstruction, ECOG > 3, comorbidities precluding safe surgery, distant metastases, massive ascites (>2 L), or preoperative imaging suggesting suboptimal cytoreduction (residual tumor ≥ 1 cm) [5,11,12].

Inclusion criteria: histology—proven or suspected stage II–IV disease, ECOG ≤ 3, and response or stable disease to NACT. Exclusion criteria included lung metastasis, ≥3 liver segments involved, progression on NACT, or intraoperative findings such as porta hepatis encasement or diffuse small bowel serosal deposits [13].

### 2.3. Surgical Evaluation and Procedures

Preoperative scoring systems (e.g., Fagotti or Peritoneal Cancer Index (PCI)) were not used. Resectability was assessed intraoperatively, usually via mini-laparotomy. If intraoperative findings suggested that optimal cytoreduction could be achieved, surgery was continued. Otherwise, the procedure was abandoned.

Bowel resections were classified as follows: colon resection; any colon segment, including rectosigmoid, small bowel resection, or composite resection; both colon and small bowel resections. We adopted the classification system described by Dr. Roberto Tozzi and cited his work accordingly. Colon resections were further categorized as total colectomy, subtotal colectomy (defined as any extra-pelvic segmental resection), and rectosigmoid resection. Additionally, patients were stratified into two groups based on the extent of bowel resection: single bowel resection versus multiple bowel resections (≥2), as previously described in the literature [4].

Preoperatively, patients did not receive any special diet or bowel preparation. The indications for bowel resection are the finding of tumor infiltration into the muscularis of the bowel and the prevention of obstruction. Bowel resection was avoided when a tumor involving serosa could be resected. The procedure of pelvic deperitonealization was performed as a whole, which can be done with the uterus by using the technique of Hudson and Chir [14].

### 2.4. Postoperative Management and Follow-Up

According to institutional policy, we followed patients who had undergone major surgery in the interim intensive care unit for observation purposes for 24 h. Patients with cardiac problems, those requiring vasopressor support, those undergoing multiple resections, or those with chest tube placement were monitored in the postoperative intensive care unit for a longer period depending on the multidisciplinary team’s decision.

Patients were followed for at least 12 months. Survival was measured from CS to death or last follow-up. Censoring was in November 2022. Median follow-up was based on censored cases as of November 2021 [15].

### 2.5. Data Collection

Continuous variables: age, BMI, CA-125, units of transfusion, number of hospitalizations. Categorical variables: ECOG, CRS type, histology, ascites, optimal vs. suboptimal cytoreduction, short- and long-term mortality, surgical procedures. Disease extent and complexity were also presented using spiderweb diagrams.

### 2.6. Outcome Definitions

Primary outcomes were 90-day mortality and overall survival. International Federation of Gynecology and Obstetrics (FIGO) criteria were used for staging, and the histology of the tumor was determined according to the World Health Organization criteria for typing ovarian neoplasms. Optimal cytoreduction and suboptimal cytoreduction were defined as macroscopic residual diseases with a maximal diameter <1 cm and ≥1 cm, respectively [3]. We cannot correctly identify the R0, 0.5 cm, and other subcategories from the chart review. However, we can identify based on the 1 cm threshold, which was consistently recorded for most patients. Platinum resistance was defined as relapse occurring within 6 months following the completion of first-line platinum-based treatment.

### 2.7. Statistical Analysis

Categorical variables were analyzed using the chi-square or Fisher’s exact test, and continuous variables were compared using the Mann–Whitney U test. Overall survival (OS) was defined as the time from the date of diagnosis to death or last follow-up. Median follow-up duration was estimated using the reverse Kaplan–Meier method. Kaplan–Meier survival analyses were generated for the entire cohort (stage II–IV) and separately for stage III and stage IV subgroups. OS was compared between patients who underwent bowel resection and those who did not using the log-rank test. Stratified survival curves by bowel resection status were plotted for each stage subgroup, with number-at-risk tables and censoring tick marks included.

Univariate Cox proportional hazards regression analyses were performed to assess the association between individual clinical and surgical variables and OS. Variables with a *p*-value < 0.10 in the univariate analysis were considered for inclusion in the multivariable model.

In order to minimize the risk of overfitting and enhance model reliability, we followed the events per variable (EPV) ≥10 criterion, as recommended for Cox proportional hazards regression modeling in clinical research [16]. According to this approach, one covariate may be included for every ten observed events. Given that 77 deaths occurred in our cohort, the final multivariable model was limited to a maximum of eight covariates. This strategy is consistent with established modeling recommendations aimed at preserving statistical power and reducing the risk of unstable estimates in moderate sized datasets.

Based on this threshold and clinical relevance, the following variables were selected for inclusion in the multivariable model:

Age (continuous).
Histological subtype (categorical: low grade serous, mucinous, mixed, transitional, carcinoid; reference: high-grade serous).
Timing of surgery (primary cytoreductive surgery vs. interval debulking surgery).
Secondary cytoreduction (yes/no).
Stoma formation (yes/no).
Residual disease status (optimal vs suboptimal).
Platinum resistance (yes/no).
Lymph node resection (yes/no).

Multicollinearity among covariates was evaluated using chi-square tests for categorical variables and variance inflation factors for continuous predictors. No significant collinearity was identified. Final model selection was based on statistical significance, clinical interpretability, and parsimony. In all regression models, binary categorical variables were coded as 0 = no and 1 = yes, with “no” serving as the reference category. For the histological subtype variable, high-grade serous carcinoma was designated as the reference group.

To further explore the potential interaction between residual disease status and bowel resection, a four-level composite variable was constructed by combining cytoreduction status (optimal vs. suboptimal) with bowel resection status (performed vs. not performed). Kaplan–Meier survival curves were generated based on this classification, and survival differences among the four groups were assessed using the log-rank test.

In a second subgroup analysis, we investigated the combined impact of surgical timing and bowel resection. Patients were classified into four groups based on the type of surgery—primary cytoreductive surgery (CRS) or interval debulking surgery (IDS)—and whether they underwent bowel resection. These groups included: primary CRS without bowel resection, primary CRS with bowel resection, IDS without bowel resection, and IDS with bowel resection. Kaplan–Meier survival curves were plotted accordingly, and differences among groups were evaluated using the log-rank test.

All statistical analyses were performed using Stata version 18.0 (StataCorp LLC, College Station, TX, USA). This version was used uniformly for all survival analyses, Cox regression models, and subgroup analyses to ensure methodological consistency, as previous analyses involved multiple statistical tools. Hazard ratios, particularly those arising from small subgroups, were interpreted with caution and not overemphasized in the final interpretation. All statistical tests were two-tailed, and *p*-values less than 0.05 were considered statistically significant unless otherwise specified.

## 3. Results

A total of 127 patients with FIGO stage II–IVB ovarian cancer who underwent CS between 1 September 2007, and 1 November 2021 were included. Among them, 99 (78%) had CS during primary surgery, while 28 (22%) underwent IDS. A total of 69 patients underwent CS without the need for bowel resection, whereas 58 (46%) required bowel resection to achieve optimal cytoreduction. Single-segment resections were performed in 46 patients, and multiple bowel resections in 12 patients (80% and 20% of all bowel resections, respectively). Among bowel resections, 85.5% were colon resections, 2.3% were small bowel only, and 12.2% were combined colon and small bowel resections. Of the colon resections, 73.2% were rectosigmoid (Hudson type II–III radical oophorectomy), 19.6% subtotal colectomy, and 7.2% total colectomy. The total stoma rate was 34% (20/58), and bowel anastomosis was performed in 46 patients (79.3%). A protective stoma was created in 28.9% (13/46) of those undergoing anastomosis.

### 3.1. Patients and Extent of Disease

Baseline characteristics of the study cohorts are summarized in Table 1.

**Figure 1:**
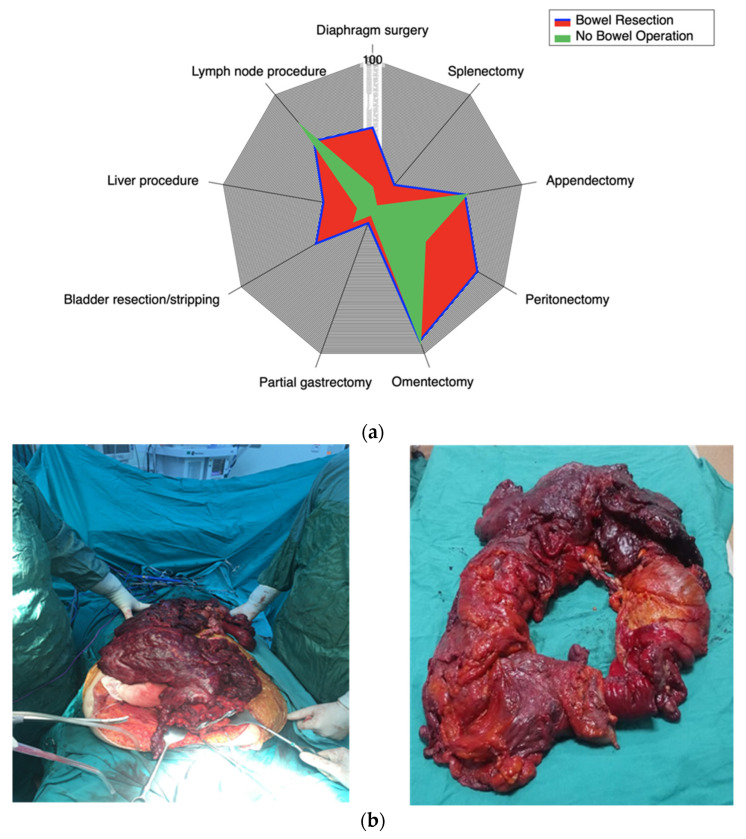
(**a**) Spider-web representation of the distribution of surgical procedures during CRS in patients with and without bowel resection. This figure visually compares the proportion of each major intraoperative step between the two cohorts. (**b**) Intraoperative and postoperative images of extensive bowel resection. (**Left**) Intraoperative view demonstrating massive tumor burden involving the bowel and surrounding tissues during CS. (**Right**) Gross specimen of the resected bowel showing the extent of tumor infiltration and resection.

All of the patient characteristics, except for the ECOG score and ascites, were similar; women undergoing bowel resection had a worse ECOG score and a higher rate of ascites (*p* < 0.05). Patients who required bowel resection also had a more extensive disease and a more complex CS. The frequency of additional surgical procedures differed significantly between the groups (Table 2). A visual summary of these interventions is presented in the spider-web diagram (Figure 1a) and intraoperative image (Figure 1b). Despite advanced disease and worse ECOG scores, optimal CS rates were similar: 89.1% vs. 83.3% (*p* = 0.653).

### 3.2. Primary Outcomes

#### 3.2.1. Clinical Outcomes

There were no intraoperative deaths in either group. The 90-day mortality rate was similar in patients with and without bowel resection (*p* = 0.411). Despite mortality being higher in the bowel resection group, it did not reach statistical significance. There was a higher number of readmissions in the bowel resection group (Table 3) within the first three months after CS among those who had undergone a bowel resection (*p* = 0.001), but this difference was not statistically significant (*p* = 0.462) after the three-month mark.

#### 3.2.2. Overall Survival Analysis

**Figure 2:**
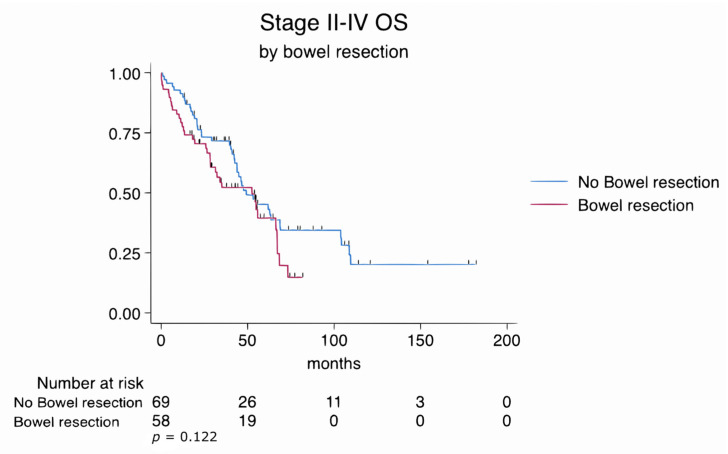
Kaplan–Meier survival estimates for all patients stratified by bowel resection status (log-rank *p* = 0.122).

Overall mortality, defined as death due to any cause during the entire follow-up period (median 46 months), was 60.3% in the bowel resection group and 60.9% in the no bowel resection group (*p* = 0.952). The overall survival rates of patients who underwent bowel resection and those who did not were comparable, with no statistically significant difference observed between the groups (log-rank *p* = 0.122) despite lower ECOG scores and more extensive disease necessitating complex surgical procedures in the bowel resection cohort (Figure 2).

**Figure 3:**
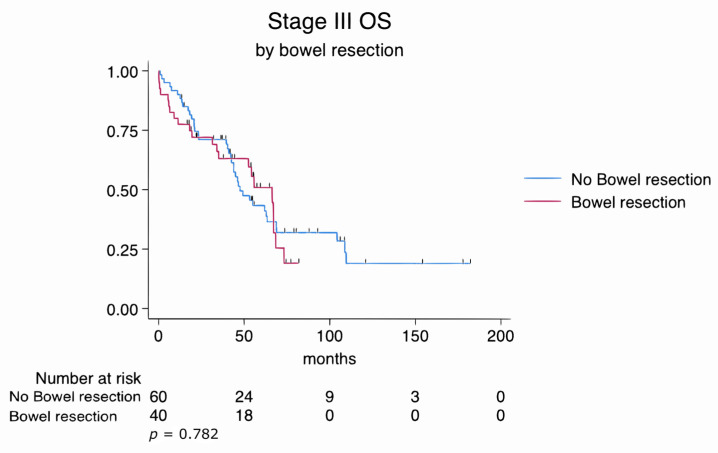
Kaplan–Meier survival estimates for FIGO stage III patients stratified by bowel resection status (log-rank *p* = 0.782).

**Figure 4:**
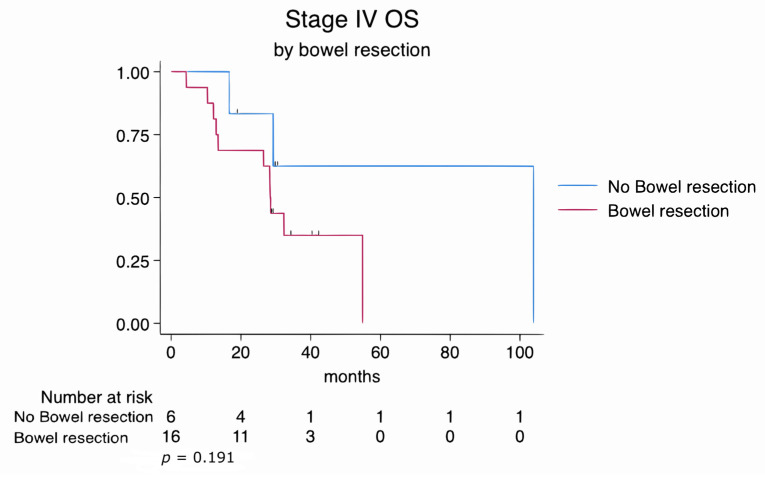
Kaplan–Meier survival estimates for FIGO stage IV patients stratified by bowel resection status (log-rank *p* = 0.191).

Similarly, subgroup analyses of FIGO stage III and stage IV patients revealed no statistically significant differences in overall survival between those who underwent bowel resection and those who did not. In stage III patients, the log-rank test yielded a *p*-value of 0.782, while in stage IV patients, it was 0.191. These findings suggest that survival outcomes were comparable regardless of the extent of disease or the complexity of the surgical procedures (Figure 3 and Figure 4).

Univariate Cox regression analysis identified several variables significantly associated with overall survival (OS) (Table 4).

Older age was associated with increased hazard (HR: 1.052, 95% CI: 1.025–1.076, *p* < 0.004). Mucinous histology demonstrated a strong association with worse OS (HR: 23.01, 95% CI: 2.00–265.0, *p* = 0.012). Patients with platinum-resistant disease also had significantly poorer survival outcomes (HR: 2.06, 95% CI: 1.12–3.79, *p* = 0.020).

Among surgical variables, lymph node dissection was significantly protective (HR: 0.337, 95% CI: 0.212–0.537, *p* < 0.001), as were secondary cytoreductive surgery (HR: 0.602, 95% CI: 0.365–0.992, *p* = 0.047) and primary cytoreductive surgery compared to interval debulking (HR: 0.565, 95% CI: 0.330–0.967, *p* = 0.037). Primary cytoreductive surgery was associated with a 43% reduction in the hazard of death compared to interval debulking surgery. Additionally, the presence of residual disease was associated with worse survival (HR: 1.855, 95% CI: 1.065–3.224, *p* = 0.029).

Bowel resection (HR: 1.438, *p* = 0.124), stoma formation (HR: 1.739, *p* = 0.080), and diaphragm stripping (HR: 1.430, *p* = 0.150) showed a trend toward increased hazard but did not reach statistical significance. Other surgical procedures, including peritonectomy, splenectomy, bladder stripping, and liver excision, were not significantly associated with OS.

Multivariate Cox regression analysis confirmed that age, type of cytoreductive surgery, and lymph node dissection were independently associated with overall survival (Table 5).

Increasing age remained a significant predictor of worse OS (HR: 1.04, 95% CI: 1.01–1.07, *p* = 0.005). Patients who underwent primary cytoreductive surgery had a significantly lower risk of death compared to those who underwent interval debulking surgery (HR: 0.54, 95% CI: 0.30–0.99, *p* = 0.047). Similarly, lymph node dissection was independently associated with improved survival outcomes (HR: 0.45, 95% CI: 0.26–0.77, *p* = 0.003).

Although mucinous histology showed a trend toward increased hazard (HR: 8.65, 95% CI: 0.68–110.43), it did not reach statistical significance (*p* = 0.097).

### 3.3. Secondary Outcomes

The transfusion rates and use of blood products were higher in the bowel resection group compared to the no bowel resection group (Table 3). Overall operation duration in patients who required bowel resection was 424 ± 38.5 min as compared to 329.5 ± 34.6 min for patients without bowel resection (*p* < 0.001). Utilization of intensive care unit was 1 day for all patients per institutional protocol and was not different between groups (*p* = 0.058).

### 3.4. Subgroup Analyses

#### 3.4.1. Subgroup Analysis Stratified by Bowel Resection

##### Residual Disease Status and Bowel Resection (Optimal vs. Suboptimal Cytoreduction)

**Figure 5:**
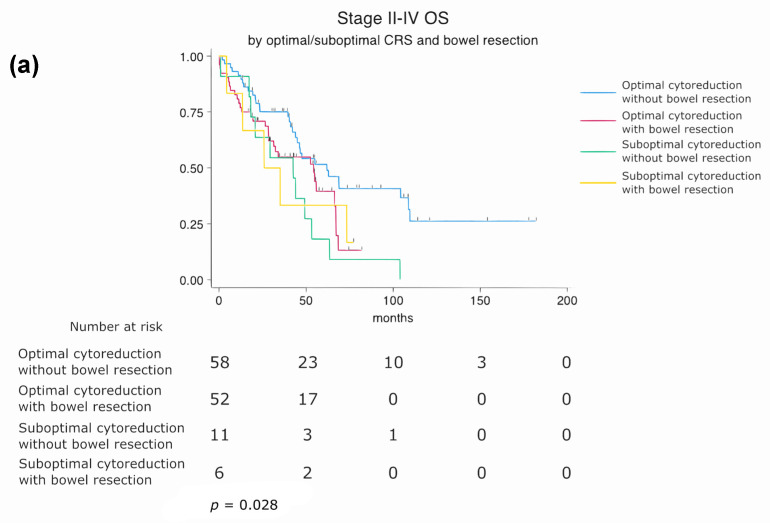

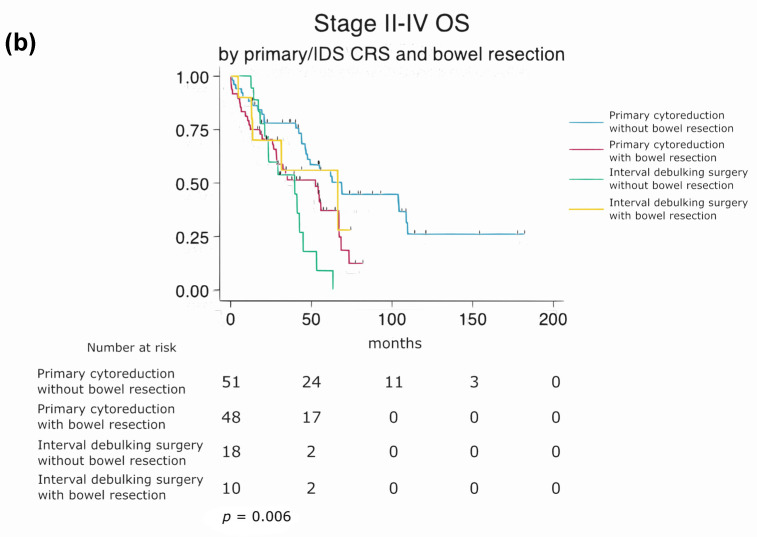
Subgroup analysis of overall survival (OS) based on cytoreduction status, surgical approach, and bowel resection. (**a**) Stratified by residual disease status and bowel resection (log-rank *p* = 0.028). (**b**) Stratified by surgical timing and bowel resection (log-rank *p* = 0.006).

Patients were stratified into four subgroups based on residual disease status (optimal vs. suboptimal cytoreduction) and bowel resection status (performed vs. not performed). Subgroup survival analysis revealed that patients who underwent optimal cytoreduction without bowel resection had the most favorable prognosis. In contrast, those with suboptimal cytoreduction and concurrent bowel resection had the poorest overall survival. A statistically significant difference in overall survival was observed between the four subgroups (log-rank *p* = 0.028), suggesting that both the completeness of cytoreduction and the need for bowel resection jointly influence long-term prognosis (Figure 5a).

##### Primary vs. IDS with and Without Bowel Resection

A second stratified subgroup analysis was performed to evaluate the combined effect of surgical timing (primary cytoreductive surgery vs. interval debulking surgery) and bowel resection. Median overall survival was 68 months for patients undergoing primary CRS without bowel resection and 52 months for those with bowel resection. In the IDS cohort, the median OS was 66 months without bowel resection and 39 months with bowel resection. Patients requiring bowel resection during IDS exhibited the worst prognosis among the four groups. A statistically significant difference in overall survival was observed between these subgroups (log-rank *p* = 0.006), indicating that the timing of surgery and the need for bowel resection may have a synergistic impact on long-term outcomes (Figure 5b).

## 4. Discussion

### 4.1. Summary of Study Results

In this study, we evaluated the impact of bowel resection during cytoreductive surgery (CS) on perioperative outcomes and overall survival (OS) in patients with advanced epithelial ovarian cancer. Although patients who underwent bowel resection had more extensive disease, lower ECOG performance scores, and a higher proportion of FIGO stage IV tumors, their OS was comparable to patients who did not require bowel resection (log-rank *p* = 0.122). Subgroup analyses by stage III and IV disease (Figure 3 and Figure 4) similarly did not show significant differences in survival between resection and non-resection cohorts.

Our results support the notion that aggressive cytoreduction, including bowel resection, can provide survival outcomes similar to patients with less extensive disease, provided optimal resection is achieved. This aligns with findings by Bacalbasa et al., who emphasized the importance of achieving R0 status to justify the increased morbidity of bowel resection [17].

In the univariate analysis, increasing age (HR = 1.05, 95% CI: 1.025–1.076, *p* < 0.004), platinum resistance (HR = 2.06, 95% CI: 1.12–3.79, *p* = 0.020), the presence of residual disease (HR = 1.855, 95% CI: 1.065–3.224, *p* = 0.029), mucinous histology (HR = 23.01, 95% CI: 2–265, *p* = 0.012), and stoma formation (HR = 1.739, 95% CI: 0.932–3.243, *p* = 0.080) were associated with worse OS. Conversely, primary cytoreductive surgery (HR = 0.565, 95% CI: 0.330–0.967, *p* = 0.037), secondary cytoreductive surgery (HR = 0.602, 95% CI: 0.365–0.992, *p* = 0.047), and lymph node dissection (HR = 0.337, 95% CI: 0.212–0.537, *p* < 0.001) were associated with improved OS. While diaphragm stripping showed a trend toward statistical significance (*p* = 0.150), other procedures such as peritonectomy, splenectomy, bladder stripping, liver resection, and disease stage (III/IV) were not significantly associated with OS. Additionally, bowel resection (HR = 1.438, 95% CI: 0.904–2.286, *p* = 0.124) did not significantly impact survival in univariate analysis.

In the multivariate Cox regression analysis, three variables remained independently associated with OS. Increasing age was confirmed as a negative prognostic factor (HR = 1.042, 95% CI: 1.010–1.070, *p* = 0.005), while lymph node dissection was associated with improved survival (HR = 0.450, 95% CI: 0.260–0.770, *p* = 0.003). Undergoing primary cytoreductive surgery was also independently associated with improved survival compared to interval debulking surgery (HR = 0.540, 95% CI: 0.300–0.990, *p* = 0.047). This corresponds to a 46% reduction in the hazard of death compared to patients who underwent interval debulking surgery. These findings reinforce the importance of surgical thoroughness and baseline patient characteristics in influencing long-term outcomes. The lack of significance for bowel resection in the adjusted model highlights that, although these patients often present with worse disease biology, the resection itself does not independently predict poorer survival.

### 4.2. Results in the Context of the Published Literature

Our findings support existing evidence suggesting that aggressive cytoreduction, including bowel resection, does not negatively impact overall survival in patients with advanced epithelial ovarian cancer when optimal cytoreduction is achieved. This is in line with Bacalbasa et al., who reported that bowel resection can contribute to improved survival by enabling complete tumor clearance (R0 resection), despite an increase in perioperative morbidity [17]. In contrast, Jaeger et al. observed poorer outcomes in patients undergoing bowel resection, although the complete cytoreduction rate in their cohort was only 28.5%—highlighting the importance of achieving R0 status in determining survival benefits [18]. Our cohort showed no significant difference in OS between patients with or without bowel resection (log-rank *p* = 0.122), supporting the importance of optimal resection over the extent of disease itself.

Lymph node dissection (LND) was one of the key prognostic factors in our multivariate analysis, independently associated with improved OS (HR = 0.450, 95% CI: 0.260–0.770, *p* = 0.003). This finding aligns with several prior studies demonstrating the survival benefit of systematic lymphadenectomy in advanced ovarian cancer. However, the LION study published by Harter et al. (2019) differed from our results [19]. While our study included patients with stage IIB–IV disease and made decisions for lymphadenectomy based on surgeon judgment and disease extent, the LION trial strictly included only patients with clinically negative lymph nodes and complete gross resection (R0), and excluded patients with bulky nodes [20]. Therefore, our results should not be interpreted as contradictory to LION but reflective of a different patient selection and real-world surgical practice. Furthermore, a 2025 subgroup analysis of the LION data focused on patients with bulky lymph nodes (stage III–IV), showing that even in such patients, the role of LND remains nuanced and might vary based on surgical feasibility and intraoperative judgment. Our inclusion of stage IIB–IV patients, with and without bulky lymph nodes, explains differences in outcomes and emphasizes the need for individualized surgical planning.

Age also emerged as an independent prognostic factor (HR = 1.042, 95% CI: 1.010–1.070, *p* = 0.005), consistent with the current literature. Previous studies have shown that increasing age is associated with decreased overall survival in epithelial ovarian cancer [21,22]. Chan et al. found that 5-year disease-specific survival dropped from 78.8% in patients under 30 to 35.3% in those over 60, highlighting the adverse prognostic role of age [23]. Liontos et al. similarly noted that older patients received less aggressive surgery and chemotherapy, which may contribute to poorer outcomes [22]. Undergoing primary cytoreductive surgery was also independently associated with improved OS (HR = 0.540, 95% CI: 0.300–0.990, *p* = 0.047), indicating a 46% reduction in the hazard of death compared to interval debulking surgery. These findings collectively support our results and emphasize the importance of considering age in surgical and therapeutic planning for epithelial ovarian cancer.

The role of stoma formation in OS remains controversial [24,25,26]. While our univariate analysis indicated a negative prognostic impact, this effect did not persist in the multivariate analysis. This is consistent with prior reports suggesting that stoma creation is more reflective of surgical complexity than an independent prognostic determinant. McNamara et al. reported worse survival in patients who required bowel resection during IDS [27]; however, our data showed no significant difference in OS or 90-day mortality in this group, possibly due to higher rates of optimal resection.

Additionally, our study did not utilize formal preoperative resectability scoring systems such as the Fagotti or PCI scores. Instead, resectability was assessed intraoperatively, usually via mini-laparotomy. While some guidelines recommend scoring systems for standardization, we found that experienced intraoperative judgment allowed the inclusion of patients who might have been deemed inoperable by such systems. This practical approach enabled successful cytoreduction in a broader patient population.

Finally, the balance between the number of neoadjuvant chemotherapy (NACT) cycles and optimal timing of surgery continues to be debated. Most of our patients underwent interval cytoreduction after 3–4 NACT cycles, with a minority receiving up to 6 due to suboptimal response. Some studies, including those by Tozzi et al. and Son et al., suggest that while prolonged NACT may reduce surgical complexity, it can also increase fibrosis and decrease R0 resection rates [6,13]. In our cohort, despite the variability in NACT cycles, optimal cytoreduction was achieved in both groups without compromising OS.

These results are consistent with those reported by Aletti et al., who demonstrated that surgical complexity did not independently influence short-term mortality, although age and performance status were crucial predictors [28]. Similarly, McNamara et al. showed that bowel resection in the setting of IDS was associated with poorer OS [27]; however, in our cohort, bowel resection did not negatively affect survival or 90-day mortality.

Bowel resection is considered a significant component of cytoreductive surgery, and the performance of bowel resection has been found to enhance the likelihood of achieving optimal cytoreduction. Several studies suggest that the potential morbidity of bowel resection is justified by its role in achieving complete cytoreduction [29].

### 4.3. Limitations and Strengths

This study has several limitations. First, its retrospective design inherently limits the ability to control for confounding variables. Specifically, due to the use of historical chart data, it was not possible to accurately classify residual disease as R0 (no gross residual), 0.5 cm, or other subcategories. Instead, residual disease was recorded based on the conventional 1 cm threshold, which was consistently documented across patients. Second, this was a single-center study, and therefore the findings may not be generalizable to institutions with different surgical practices or patient demographics. Differences in surgical expertise, institutional protocols, and patient demographics could influence outcomes. A multicenter study would help validate these findings and provide a more robust assessment of bowel resection in the context of cytoreductive surgery for advanced ovarian cancer. Recent findings by Kim et al. (2024) suggest that perioperative outcomes in bowel resections for advanced ovarian cancer were not significantly affected by the surgeon’s specialty (gynecologic oncologist vs. general surgeon), highlighting the importance of institutional surgical protocols and multidisciplinary team planning [30].

Additionally, the absence of preoperative scoring systems such as the Fagotti or PCI scores limits comparability with other studies and introduces potential selection bias in surgical decision-making. The lack of detailed molecular profiling, such as BRCA or HRD status, may also have impacted the prognostic assessment and limits translational interpretation of the findings.

Due to the retrospective nature of our study and limitations in historical data documentation, we were unable to accurately classify residual disease beyond the conventional 1 cm threshold. This limitation restricts our ability to assess the prognostic impact of residual disease size, which has been demonstrated in previous studies to be a significant factor influencing survival outcomes in advanced epithelial ovarian cancer [31].

Despite these limitations, this study has several strengths. First, it includes a relatively large patient cohort with advanced-stage epithelial ovarian cancer, treated in a high-volume center with consistent surgical protocols. Second, the study features a median follow-up of over 46 months, allowing for a robust analysis of long-term survival outcomes. Third, the surgical data are highly detailed, including stratification by bowel resection type, anastomosis, and stoma status, offering granular insights into operative complexity. Finally, the study applies both univariate and multivariate statistical methods, with supplementary Kaplan–Meier analyses stratified by stage, addressing several key methodological expectations in surgical oncology literature.

## 5. Conclusions

Bowel resection performed during CS for advanced epithelial ovarian cancer does not negatively impact overall survival when optimal cytoreduction is achieved. Despite its association with increased perioperative complexity, including higher transfusion rates and longer operative time, bowel resection was not an independent predictor of short-term or long-term mortality. Our findings suggest that bowel resection can be safely integrated into individualized surgical strategies for selected patients with extensive disease. The prognostic importance of primary debulking surgery and lymph node dissection was reinforced, while the extent and type of bowel resection did not significantly alter survival outcomes. Future prospective, multicenter studies are warranted to further validate these results and guide decision-making in complex ovarian cancer surgeries.

## Figures and Tables

**Table 1:** Demographic and clinical characteristics of the study population.

Variable	Bowel Resection (*n* = 58)	No Bowel Resection (*n* = 69)	*p*-Value
IDS/Primary CS	10/48 (17.2%/82.8%)	18/51 (26.1%/73.9%)	0.231
Optimal cytoreduction rate	52/58 (89.1%)	58/69 (83.3%)	0.653
Age (median, range)	59 (35–88)	60 (40–88)	0.433
BMI (median, range)	27.9 (14.7–40.1)	28 (16.2–44.8)	0.924
ECOG score			0.005
- 0	14/58 (24.1%)	30/69 (43.5%)	0.045
- 1	26/58 (44.8%)	33/69 (47.8%)	0.930
- 2	10/58 (17.2%)	5/69 (7.2%)	0.082
- 3	8/58 (13.8%)	1/69 (1.4%)	0.014
FIGO Stage			0.020
- II	2/58 (3.4%)	3/69 (4.3%)	0.795
- III	40/58 (69.0%)	60/69 (87.0%)	0.014
- IV	16/58 (27.6%)	6/69 (8.7%)	0.005
Histology			0.602
- High-grade serous	55/58 (94.8%)	59/69 (85.5%)	0.084
- Low-grade serous	1/58 (1.7%)	3/69 (4.3%)	0.399
- Clear cell	1/58 (1.7%)	2/69 (2.9%)	0.664
- Mucinous	1/58 (1.7%)	1/69 (1.4%)	0.901
- Transitional	0/58 (0%)	1/69 (1.4%)	0.357
- Mixed	0/58 (0%)	2/69 (2.9%)	0.191
- Other	0/58 (0%)	1/69 (1.4%)	0.357
Preoperative CA-125 (median, range)	726.8 (9.2–11476)	673.8 (4–7337)	0.622
Presence of ascites	36/58 (62.1%)	25/69 (36.2%)	0.004
Stoma rate	20/58 (34.5%)	N.A.	—
Anastomosis rate	46/58 (79.3%)	N.A.	—
Protective stoma rate	13/46 (28.9%)	N.A.	—

N.A.: Not applicable. Percentages may not total 100% due to rounding.

**Table 2:** Distribution of surgical procedures performed during cytoreductive surgery by bowel resection status.

Procedures During CS	Bowel Resection (*n* = 58)	No Bowel Resection (*n* = 69)	*p*-Value
Lymph node procedure	35 (60.3%)	51 (73.9%)	0.103
Diaphragm Stripping	32 (55.2%)	11 (15.9%)	<0.001
Splenectomy	13 (22.4%)	3 (4.3%)	0.002
Appendectomy	36 (62.1%)	44 (63.8%)	0.843
Peritonectomy	46 (79.3%)	29 (42%)	<0.001
Omentectomy	53 (91.4%)	64 (92.8%)	0.775
Partial Gastrectomy	5 (8.6%)	2 (2.9%)	0.159
Bladder Resection/Stripping	25 (43.1%)	10 (14.5%)	<0.001
Liver Procedure	19 (32.8%)	7 (10.1%)	0.002

**Table 3:** Operative and long-term outcomes of patients with and without bowel resection during CS.

Short-Term Outcomes	Bowel Resection (*n* = 58)	No Bowel Resection (*n* = 69)	*p*-Value
Mortality in 90 days	4 (6.9%)	2 (2.9%)	0.411
Rate of FFP transfusion	46 (78.6%)	20 (28.8%)	<0.001
Number of used intraoperative FFP (median, range)	2 (1–5)	1 (1–3)	0.008
Rate of red blood cell transfusion	46 (80%)	28 (40.9%)	<0.001
Number of used red blood cell packages	2 (1–4)	1 (1–5)	0.003
Rate of ICU admission	44 (75.9%)	20 (29%)	<0.001
ICU duration (day) (median, range)	1 (1–18)	1 (1–24)	0.058
Operation duration (minutes)	424 ± 38.5	329.5 ± 34.6	<0.001
**Long-term Outcomes**	**Bowel Resection** **(*n* = 58)**	**No Bowel Resection** **(*n* = 69)**	***p*-value**
Overall mortality during follow-up	35 (60.3%)	42 (60.9%)	0.952
Development of platinum resistance	7 (12.1%)	8 (11.6%)	0.934
Secondary CS during follow-up	11 (19%)	26 (37.7%)	0.021
Re-admission rate in 3 months	23 (39.7%)	9 (13%)	0.001
Number of hospital Re-admissions per patient in 3 months (median, range)	0 (0–4)	0 (0–6)	0.001
Re-admission rate in 3-6 months	10 (17.2%)	8 (11.6%)	0.363
Number of hospital Re-admissions per patient in 3–6 months	0 (0–3)	0 (0–7)	0.462

FFP: Fresh frozen plasma. ICU: Intensive care unit. CS: Cytoreductive surgery.

**Table 4:** Univariate Cox regression analysis of factors associated with overall survival.

Variable	Hazard Ratio	95% CI	*p*-Value
Age	1.05	1.025–1.076	<0.004
Bowel Resection	1.438	0.904–2.286	0.124
Secondary cytoreduction	0.602	0.365–0.992	0.047
Histology mucinous	23.01	2–265	0.012
Stage III	1.382	0.335–5.701	0.654
Stage IV	2.465	0.551–11.027	0.258
Primary CS	0.565	0.330–0.967	0.037
Stoma	1.739	0.932–3.243	0.080
Lymph node dissection	0.337	0.212–0.537	<0.001
Platinum Resistance	2.06	1.12–3.79	0.020
Diaphragm Stripping	1.43	0.887–2.347	0.150
Peritonectomy	0.795	0.508–1.245	0.317
Splenectomy	1.082	0.538–2.176	0.824
Bladder Stripping	0.877	0.486–1.586	0.664
Liver Excision	1.064	0.881–1.948	0.840
Residual Disease	1.855	1.065–3.224	0.029

Note: Reference categories for categorical variables were as follows: Surgical Timing = Interval Debulking Surgery (IDS); Bowel Resection = No; Secondary Cytoreduction = No; Histology = High-grade serous; Stage = Stage II; Stoma = No; Lymph Node Dissection = No; Platinum Resistance = No; Diaphragm Stripping = No; Peritonectomy = No; Splenectomy = No; Bladder Stripping = No; Liver Excision = No; Residual Disease = Optimal.

**Table 5:** Multivariate Cox regression analysis of factors independently associated with overall survival.

Variable	Hazard Ratio	95% CI	*p*-Value
Age	1.042	1.01–1.07	0.005
Lymph node dissection	0.45	0.26–0.77	0.003
Primary CS (vs IDS)	0.54	0.30–0.99	0.047
Mucinous histology	8.65	0.68–110.43	0.097

Note: Reference categories for categorical variables were as follows: Surgical Timing = Interval Debulking Surgery (IDS); Histology = High-grade serous; Lymph Node Dissection = No.

## Data Availability

The data that support the findings of this study are available from the corresponding author upon reasonable request.

## Abbreviations

The following abbreviations are used in this manuscript:

CS	Cytoreductive surgery
IDS	Interval debulking surgery
OS	Overall survival
EOC	Epithelial ovarian cancer
FIGO	International Federation of Gynecology and Obstetrics
ECOG	Eastern Cooperative Oncology Group
NACT	Neoadjuvant chemotherapy
PDS	Primary debulking surgery
ICU	Intensive care unit
FFP	Fresh frozen plasma
R0	No gross residual tumor
ASA	American Society of Anesthesiologists
